# Case Report: Generalized motor tonic seizures characterized by paroxysmal fast activity on EEG in a Tonkinese cat

**DOI:** 10.3389/fvets.2025.1597258

**Published:** 2025-09-16

**Authors:** Laura Brewińska, Paulina Drobot, Marcin Wrzosek

**Affiliations:** ^1^Department of Internal Diseases with a Clinic for Horses, Dogs, and Cats, Faculty of Veterinary Medicine, Wrocław University of Environmental and Life Sciences, Wrocław, Poland; ^2^Neuroteam Veterinary Specialist Clinic, Wrocław, Poland; ^3^VetImaging Computed Tomography, Wrocław, Poland

**Keywords:** feline epilepsy, tonic epilepsy, electroencephalography, paroxysmal fast activity, phenytoin

## Abstract

Epilepsy is one of the most common neurological disorders in cats, affecting 1%−2% of the feline population. Feline epilepsy can often be managed with standard anti-seizure medications, which typically offer moderate to good seizure control. However, a small subset of cats may experience drug-resistant epilepsy and require alternative treatment options. The scientific understanding of the mechanisms underlying feline epilepsy has not yet reached the applicability seen in human studies. A deeper understanding of feline epilepsy will aid in developing effective treatment strategies. Electroencephalography (EEG) is an important tool for extending veterinary epilepsy classification. In the presented case, the disease was classified as idiopathic epilepsy with a Tier III confidence level according to the International Veterinary Epilepsy Task Force classification. The seizures were further characterized as primarily generalized tonic, with occasional focal seizures also observed, and the condition met the criteria for drug-resistant epilepsy. EEG findings revealed typical spike, sharp and slow wave, and polyspike activity, along with atypical paroxysmal fast activity. Non-standard treatment with phenytoin demonstrated potential efficacy in this case.

## 1 Introduction

Seizures are one of the most common neurologic disorders of cats, affecting ~1%−2% of the general feline population (1). As proposed by International Veterinary Epilepsy Task Force (IVETF), I confidence level for the diagnosis of idiopathic epilepsy is based on a history of two or more unprovoked epileptic seizures occurring at least 24 h apart, age at epileptic seizure onset of between 6 months and 6 years, unremarkable inter-ictal physical and neurological examination, and no significant abnormalities on minimum data base blood tests and urinalysis. Tier II confidence level for the diagnosis of IE is based on the factors listed in tier I and unremarkable fasting and post-prandial bile acids, magnetic resonance imaging (MRI) of the brain (based on an epilepsy-specific brain MRI protocol) and cerebrospinal fluid (CSF) analysis. Tier III confidence level for the diagnosis of IE is based on the factors listed in tier I and II and identification of electroencephalographic abnormalities characteristic for seizure disorders (2). The aforementioned scheme was initially proposed for canine patients, but could be implemented in feline ones. While once primary [idiopathic] epilepsy was considered rare or nonexistent in cats, it is now recognized more frequently, albeit at a lower incidence compared to dogs. Due to high prevalence of structural epilepsy in felines, a thorough diagnostic workup is recommended for all cats undergoing initial evaluation for seizures (3). Although generalized tonic or tonic-clonic seizures can occur, focal seizures are more commonly observed in cats than in dogs. The most widely described focal seizure manifestation in felines is complex partial cluster seizures with orofacial involvement (4, 5).

By definition, a motor seizure involves skeletal musculature resulting in any phenotypic manifestation. It consist of an increase (positive) or decrease (negative) in muscle contraction to produce a movement. A tonic seizure is a type of motor seizure characterized by unilateral or bilateral limb stiffening or elevation, often with neck stiffening (6). This stiffening results from sustained increased muscle contraction and lasts from a few seconds to several minutes. Tonic seizures can be focal, affecting a single limb, or generalized, involving bilateral motor activity. They result in versive or dystonic movements (7).

In the presented case, seizures were classified as idiopathic epilepsy (epilepsy of unknown cause), manifesting primarily as motor, generalized tonic (dystonic) seizures with a generalized onset confirmed by EEG with the Tier III confidence level. In human medicine, an extensive classification system for seizures, as established by the International League Against Epilepsy (ILAE), informs treatment decisions (8). However, in veterinary neurology, the scientific understanding of epilepsy mechanisms in companion animals remains underdeveloped compared to human studies (7). One contributing factor is the infrequent use of electroencephalographic (EEG) examinations. Feline epilepsy is typically managed with standard anti-epileptic drugs (AEDs), such as phenobarbital, levetiracetam, and zonisamide, which provide generally moderate to good seizure control (3). Nevertheless, a subset of cats with drug-resistant epilepsy may require alternative treatment options, such as epilepsy surgery or off-label medications. Accumulation of further relevant data will aid in guiding the treatment strategy (9). This highlights the importance of publications detailing fully documented cases with atypical presentations or responses to treatment such as the one described here.

## 2 Case description

A 6-year-old, castrated male Tonkinese cat was referred for neurological consultation due to drug-resistant epilepsy. Three years prior, the cat experienced its first seizure episode, which progressed into cluster seizures lasting for 2 weeks. The cat was initially treated with antibiotics, although the specific type was not documented, and experienced spontaneous improvement. One year later, recurrent seizures began, characterized by episodic behavioral arrest, balance disturbances with increased limb tonus, and sporadic blinking, whisker twitching, and vocalization. No autonomic signs or loss of consciousness were observed. The patient experienced dozens of episodes daily, lasting a few seconds with varying frequency. According to the owner, the cat had always exhibited behavioral abnormalities, including excitability, nocturnal restlessness, and irritability, which worsened as epilepsy symptoms progressed. At the time of presentation, the cat also displayed excessive licking, leading to bald patches. The pre-referral diagnostic work-up and treatment included a complete blood count (CBC) and serum biochemistry, which were unremarkable except for occasional slight increases in creatinine and blood urea nitrogen (BUN). Fasting blood ammonia and the bile acids stimulation test were within normal limits. Serological tests for FIV/FeLV and *Toxoplasma gondii* (IgM and IgG titers) were negative. *Bartonella henselae* IgM was positive, while IgG was borderline. *Borrelia burgdorferi* IgG was positive, with borderline IgM. Systolic blood pressure measurements were within normal limits. Low-field MRI showed no abnormalities, and CSF analysis was normal. Several antiepileptic drugs (AEDs) were prescribed, including phenobarbital, zonisamide, levetiracetam, gabapentin, and CBD oil. After the administration of CBD oil, improvement was observed for 1 week. No improvement was noted with zonisamide or levetiracetam treatment. Phenobarbital administration did not result in any improvement, but it led to a marked elevation in liver enzymes, prompting its discontinuation. The current medication regimen included CBD oil (15 mg/kg TID), gabapentin (30 mg/kg TID), and trazodone (10 mg/kg SID), the latter prescribed due to the aforementioned behavioral abnormalities.

A clinical examination was unremarkable. A neurological examination revealed normal consciousness, posture, and gait. Cranial nerve function, spinal reflexes, and proprioception were also unremarkable. However, frequent involuntary movements were observed, including sudden body stiffening accompanied by lip-smacking and eye-blinking, each lasting few seconds only.

**Figure 1:**
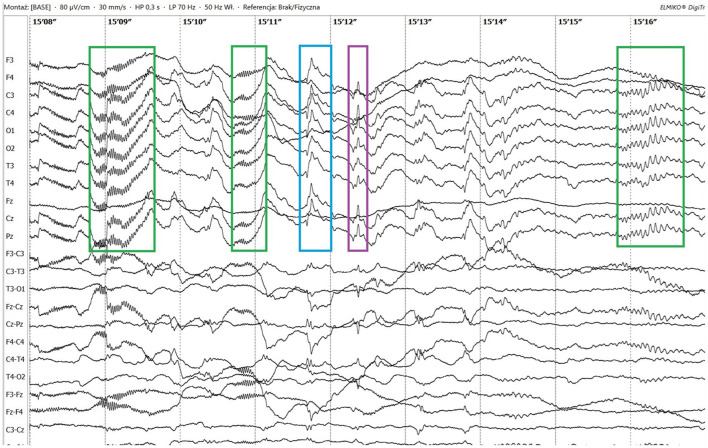
Ictal paroxysmal fast activity (green boxes), sharp wave (blue box) and spike (purple box).

**Figure 2:**
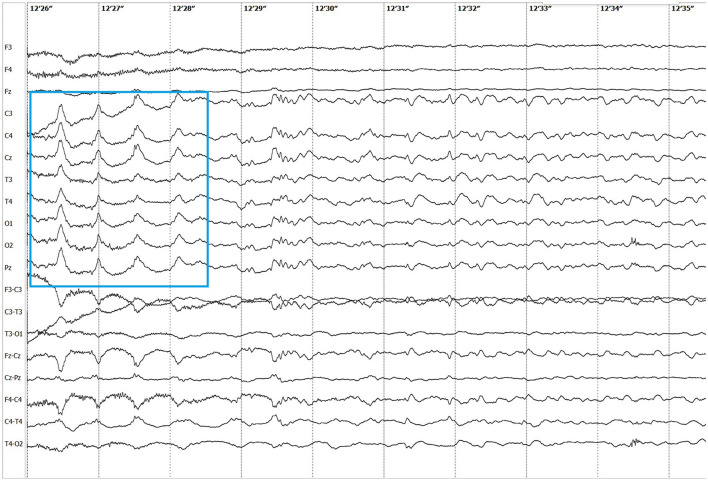
Post-ictal slow wave activity (1,5 Hz).

**Figure 3:**
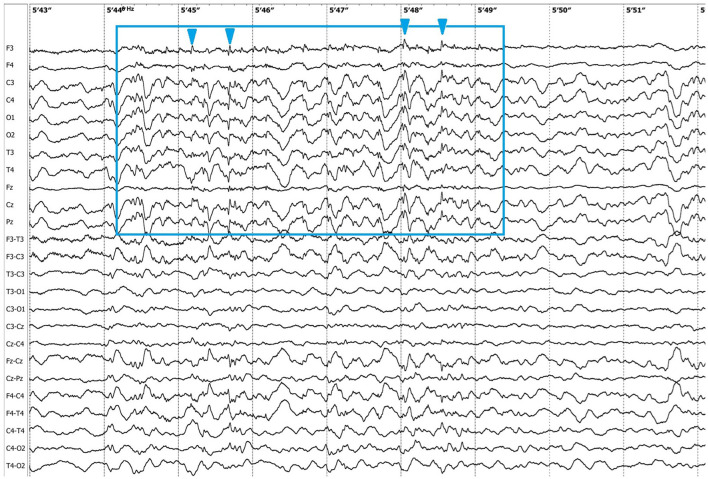
Photic-driven responses (blue arrowheads) during photic stimulation (6 Hz).

Electroencephalographic (EEG) examination was conducted using movEEG ExG32 DigiTrack Elmiko Biosignals electroencephalographic equipment and EEG DigiTrack software (version 15.1). Needle electrodes were utilized in an 11-channel reference montage (F3, F4, Fz, C3, C4, Cz, T3, T4, Pz, O1, O2, -Ref.), with the reference electrode placed on the nose. The patient was premedicated with medetomidine, administered at a dose of 20 μg/kg into the quadriceps femoris muscle. The whole examination was performed under sedation, medetomidine has not been antagonized. Background activity exhibited a dominant delta rhythm. The EEG revealed multiple epileptiform discharges characterized by interictal sharp waves, spikes and polyspikes predominantly in the central leads, with greater intensity on the left side (C3 derivation). Ictal seizure activity was highly atypical and consisted not only of aforementioned superimposed transients and but also paroxysmal fast activity (PFA) (Figure 1). In the post-ictal phase, generalized slow (~1,5 Hz) wave activity could be appreciated (Figure 2). The PFA had low amplitude and a very high frequency (>30 Hz in most cases). These discharges lasted for 0.5–1.25 s and recurred every 1.5–2 s. The series began with very low amplitude, which progressively increased with each subsequent series of spikes, ultimately leading to visible seizures on the video-EEG. After the peak activity, the spikes subsided, and the EEG returned to background activity until the next episode occurred. Photic stimulation did not significantly increase epileptiform activity, although one seizure coincided with the onset of photic stimulation. Normal photic-driven responses were recorded during photic stimulation (10) (Figure 3). The video-EEG recording showed that the epileptic discharges were accompanied by tonic convulsions of the entire body, with twenty-four seizures observed during the study period, which lasted 30 min. A few ocular and movement artifacts were noted. Due to cluster seizures, midazolam (0,2 mg/kg) was administered intravenously during examination what resulted in in absence of visible seizures on the video, but have not had any influence on PFA intensity.

**Figure 4:**
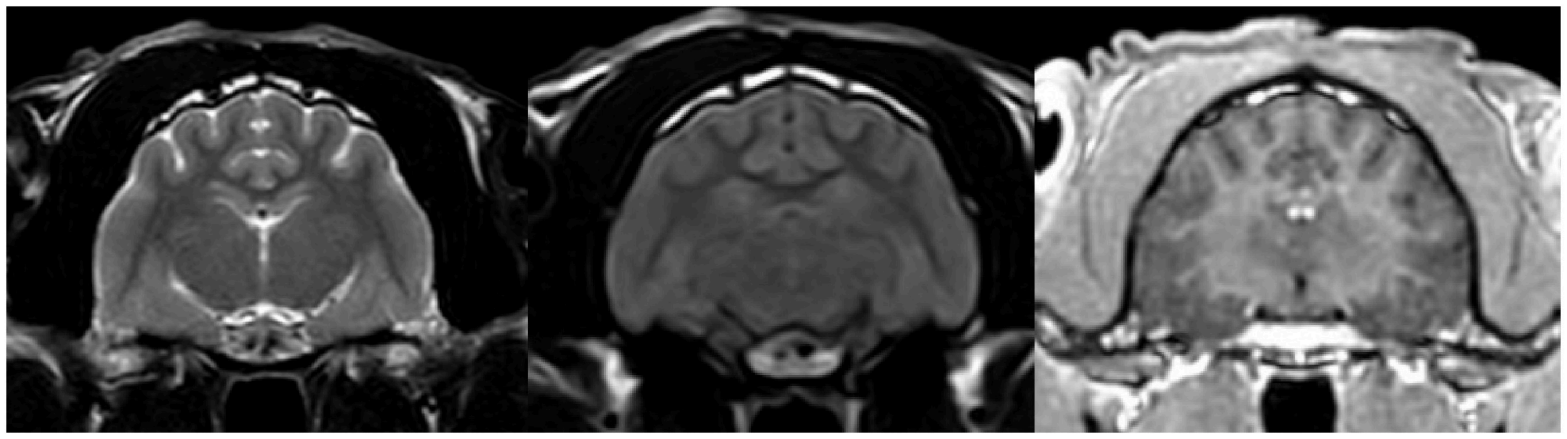
MRI transverse section of the brain at the level of hippocampus in T2W, FLAIR and T1W post contrast sequences respectively. The images show no abnormalities.

After reviewing the EEG results, topiramate (10 mg/kg BID) treatment was prescribed for the next 3 weeks; however, the cat continued to experience cluster seizures daily. At this point, phenytoin was prescribed (3 mg/kg SID), which resulted in a marked reduction in seizure frequency. The patient experienced one seizure every few days. Nevertheless, due to severe anorexia, the drug had to be discontinued, which resulted in a moderate recurrence of seizures (one to several seizures most days). However, anorexia persisted, leading to moderate cachexia. Discontinuation of trazodone resulted in an increase in appetite. A follow-up high-field MRI of the brain (Figure 4) as well as CSF examinations were unremarkable.

At this point, electrodiagnostic examination (EDX) was performed using Nicolet Viasys Healthcare portable electrodiagnostic equipment with version 11.0 of the Viking Quest software. Prior to testing, the cat was premedicated with butorphanol at a dose of 0.4 mg/kg, administered IV. General anesthesia was induced using propofol at an initial dose of 2 mg/kg and a maintenance dose of 0.4 mg/kg/min. Motor nerve conduction velocity of the left sciatic/tibial nerve showed normal compound muscle action potential morphology and amplitude (20.5–21.1 mV), with normal conduction velocity (73–112 m/s). A repetitive nerve stimulation test performed at 2 Hz frequency demonstrated a 2% decrement, indicating normal neuromuscular junction function. F-wave latency was within the normal range, so the F ratio was not calculated. Electromyography (EMG) revealed no pathological potentials in the paraxial, paraspinal, or head muscles. EMG was conducted twice during the examination—for the first time, during general anesthesia and for the second time while the cat was recovering from anesthesia to minimalize propofol's influence on the muscle tone. In conclusion, EDX findings were within normal limits. Phenytoin was re-administered at the same dose.

At the follow-up 1 month later, the cat was seizure-free. The cat's caretaker insisted on gradually tapering off phenytoin due to concerns about side effects (anorexia), which unfortunately resulted in the recurrence of seizures teo month later. For another month, the cat remained seizure-free at a dose of 3 mg/kg every other day, but then again the seizure occured Finally, the effective dose of phenytoin was established at 3 mg/kg SID. At this dosage, the cat remained seizure-free, and its appetite was maintained at a slightly reduced but adequate level to support normal weight. Last follow-up was performed one and a half year later and the cat was seizure free on phenytoin treatment.

## 3 Case outcome

Treatment with phenytoin markedly improved the patient's quality of life, both in terms of seizure control and behavioral abnormalities. The cat was seizure-free and stopped exhibiting abnormal excitability, restlessness, as well as compulsive licking. Discontinuation of trazodone and CBD oil was possible without the recurrence of clinical signs, as well as a reduction of gabapentin to 10 mg/kg SID. Blood test results remained normal during follow-ups. The main complaint regarding the treatment was anorexia, which was severe at times, but finally appetite stabilized, and it was possible to maintain the cat's body condition at a 4/9 score during the treatment.

## 4 Discussion

By definition, absolute confirmation of the epileptic nature of an event can be obtained by simultaneously observing the characteristic EEG changes and the physical manifestation of seizures (2). Thus, the presence of epileptic episodes in the patient could be definitively confirmed based on the EEG results. However, due to the tonic nature of the events and a favorable response to phenytoin treatment (which has been shown to be effective not only against epileptic activity but also for central and peripheral muscle spasticity and neuromyotonia in cats) (11–13), a myotonic component was suspected. It was hypothesized that the high-amplitude polyspikes captured on EEG could, in fact, be muscle artifacts caused by myotonic contraction of the temporal muscles. Although midazolam administration led to the cessation of overt convulsive activity, epileptiform discharges persisted on EEG. Residual muscle tone may have contributed to artifacts, potentially complicating interpretation. To exclude this possibility, EDX were performed. As the findings were within normal limits, neuromuscular involvement was excluded, and muscle artifact was considered an unlikely cause of the observed PFA.

The presented feline case shares some similarities with human Lennox-Gastaut syndrome (LGS). LGS is a drug-resistant epilepsy syndrome that usually begins in childhood (less commonly in adolescence or adulthood), with psychological and physical comorbidities being common. It is characterized by different types of seizures, predominantly tonic seizures. It is not uncommon for a single patient to experience more than one type of seizure, often both focal and generalized (14, 15). LGS has a typical EEG pattern, which consists of high-amplitude PFAs, followed by generalized slow waves as well as polyspikes and spike and wave complexes (15). PFA is characterized by paroxysmal EEG events with alpha, beta, and gamma frequencies (8 Hz up to >30 Hz), an amplitude higher than baseline activity, a duration of at least 0.2 s, and a higher occurrence during NREM sleep. Despite being a highly characteristic key feature, PFAs are not pathognomonic for LGS and can be found in other brain conditions (16).

There are several similarities and differences between LGS and this case. The seizure types observed in this patient included two distinct forms: primarily generalized tonic seizures, which are highly characteristic of LGS, and less frequently focal seizures (earlier described in literature as complex partial cluster seizures with orofacial involvement). The disease was classified as drug-resistant since appropriately selected standard antiepileptic drugs (AEDs), administered at therapeutic and maximally tolerated doses, failed to control seizure activity (17). Additionally, the EEG pattern showed PFA alongside polyspikes, spikes, slow and sharp waves, with a marked increase in seizure frequency following medetomidine premedication. Medetomidine influences EEG background activity by increasing delta and theta band frequencies, which are also typical of NREM sleep in cats (10, 18). This may have contributed to the intensification of seizure activity observed during EEG. Similar use of α2-agonists has been reported in humans; dexmedetomidine enabled reliable detection of PFA during intraoperative EEG in patients with LGS (19). Despite these similarities, key differences were noted. In our case, the frontal dominance commonly reported in patients with LGS was not observed. Interictal slow wave epileptiform activity (Figure 2) was present but did not include spike and wave complexes, which are one of the mandatory features in LGS diagnosis in humans. Unfortunately, the EEG was performed under sedation so background activity in the awake state—where abnormalities are often noted in affected human patients—could not be assessed. In humans, LGS is frequently associated with behavioral comorbidities and cognitive dysfunction. In the presented feline case, behavioral abnormalities were noted but resolved following anti-seizure therapy, suggesting that these signs were more likely a consequence of uncontrolled seizure activity rather than an independent comorbidity (20). A major distinction was the adult-onset nature of the condition. In humans, late-onset LGS can sometimes be associated with prior brain injury of varying etiologies (21). *B. burgdorferi* and *B. henselae* seropositivity are not known to be associated with seizures in felines (22, 23), but pre-existing encephalitis, such as feline hippocampal necrosis, could not be ruled out entirely in this case. Moreover, the outcome in this case was excellent, whereas the prognosis for LGS in humans is typically poor. The condition in this patient responded well to phenytoin, whereas the efficacy of phenytoin in LGS varies. Phenytoin exerts its effects by blocking voltage-dependent sodium channels, which are responsible for sustaining the action potential. This mechanism interrupts the positive feedback loop that drives high-frequency repetitive firing, thereby preventing the spread of the seizure focus (8). In patients with LGG it may be used during seizure exacerbations; however, chronic use is contraindicated due to its potential to worsen atypical absence and myoclonic seizures (21). In this case, there were no evident alterations in consciousness or generalized myoclonus, which may have contributed to the drug's efficacy. It is also noteworthy that feline phenytoin metabolism differs significantly from that of humans; it is slower and does not result in a long-term decline in plasma concentrations (24). Unfortunately, phenytoin plasma concentration could not be determined in this patient due to the unavailability of commercial tests.

Given these considerations, this case shares partial phenotypic overlap with LGS but does not meet all the criteria for a direct analog. Instead, it may represent a feline epilepsy syndrome with LGS-like features, particularly in EEG and seizure semiology, rather than a direct equivalent. The comparison to LGS is therefore illustrative but not definitive.

## 5 Conclusion

This case describes an unusual form of epilepsy in a cat, with both focal and generalized seizures that became more pronounced during delta activity of the brain under medetomidine sedation, abnormal EEG findings, and resistance to standard anti-seizure medications. Seizure control was achieved only after starting phenytoin, a drug rarely used in cats. The drug was generally well-tolerated, with only minor adverse effects (transient decrease of the appetite) being noted. This outcome suggests that phenytoin may be a useful option in selected cases of drug-resistant epilepsy, and its potential role deserves further evaluation.

The EEG findings were essential in confirming the diagnosis and understanding the seizure pattern, especially when clinical signs were ambiguous. This case highlights the value of EEG as a diagnostic tool in veterinary neurology and shows how it can support treatment decisions.

While some features were similar to epilepsy syndromes described in humans, such comparisons should be made cautiously. The main goal of this report is to expand understanding of feline epilepsy and encourage further investigation into treatment options.

## Data Availability

The raw data supporting the conclusions of this article will be made available by the authors, without undue reservation.

## Supplementary material

Supplementary Figure 1 Timeline of the case.

Supplementary Video 1 Recording provided by the cat's owners presenting seizure.

Supplementary Video 2 Video-EEG during tonic seizure.
