# Supplementary material for: Case Report: Generalized motor tonic seizures characterized by paroxysmal fast activity on EEG in a Tonkinese cat
Source: Front Vet Sci. 2025 Sep 16;12:1597258. doi: 10.3389/fvets.2025.1597258 (PMC12481607; doi:10.3389/fvets.2025.1597258)
Supplement: Supplementary Figure 1 — Timeline of the case. [file Data_Sheet_1.pdf]

Timeline of the case

2021-07-01

The first seizure occurred as multiple clusters of tonic seizures (occasionally with orofacial involvement) over 2 weeks.

2022-07-01

Recurrence and continuation of seizures in clusters.

2023-03-14

Pre-referral treatment: phenobarbital 2.5 mg/kg BID (discontinued due to marked elevation of liver enzymes), levetiracetam 20 mg/kg TID, zonisamide 10 mg/kg BID, prednisolone 1 mg/kg SID, CBD 15 mg/kg TID, gabapentin 30 mg/kg TID, trazodone 10 mg/kg SID.

2023-03-15

Pre-referral workup: CBC, fasting ammonia, SAA, bile acid stimulation test, blood pressure, low-field brain MRI, CSF unremarkable; FIV/FeLV - AG negative, T. gondii - AB negative; B. henselae - IgM positive, B. burgdorferi - IgG positive, IgM borderline.

2024-03-17

Referral due to drug-resistant epilepsy. Treatment included CBD 15 mg/kg TID, gabapentin 30 mg/kg TID, trazodone 10 mg/kg SID. Despite multiple tonic and orofacial seizures during consultation, neurological exam was normal.

2024-03-18

Video-EEG revealed generalized polyspike activity, capturing 24 tonic seizures in 30 minutes. Thiamine serum concentration was normal. MPS VI PCR was normal.

2024-03-18

Initiation of topiramate at 5 mg/kg BID resulted in worsening of clinical signs, with tonic seizures occurring multiple times per hour.

2024-05-14

Control high-field brain MRI and CSF PCRs (T. gondii, B. henselae, coronavirus, bornavirus) were unremarkable. Phenytoin initiated at 3 mg/kg SID.

2024-05-27

Marked reduction in seizure frequency and intensity to one seizure every few days, but the cat developed severe anorexia.

2024-06-10

Discontinuation of phenytoin due to anorexia led to increased seizure frequency (1–several/day). Anorexia persisted. Stopping trazodone improved appetite.

2024-07-02

Electrodiagnostic tests (EMG, ENG, RNS) were within normal limits. Phenytoin re-administered.

2024-08-03

Very good quality of life with complete seizure freedom, no anorexia, and reduced excitability. Phenytoin dose gradually reduced.

2024-10-02

Control bloodwork within normal limits. Seizures recurred during phenytoin taper. Re-administration at 3 mg/kg SID caused anorexia. Effective dose: 3 mg/kg every other day.

2024-11-10

Seizure recurrence likely due to GI inflammation and malabsorption. Phenytoin increased to 3 mg/kg SID. Complete seizure control with slight appetite decrease.

2025-03-15

Phenytoin maintained at 3 mg/kg SID. Very good quality of life, seizure-free, no anorexia (BCS 4/9).
